# Clinical trial evaluating the efficacy and safety of percutaneous prostate cancer lesion-targeted microwave tissue coagulation for prostate functional preservation: MicroPro2

**DOI:** 10.1097/SP9.0000000000000051

**Published:** 2025-06-18

**Authors:** Atsuko Fujihara, Takumi Shiraishi, Go Horiguchi, Takashi Ueda, Masatsugu Miyashita, Yuta Inoue, Yayoi Iwami, Akari Naito, Satoshi Teramukai, Toshiko Ito-Ihara, Osamu Ukimura

**Affiliations:** a Department of Urology, Kyoto Prefectural University of Medicine, Kyoto, Japan; b Department of Biostatistics, Kyoto Prefectural University of Medicine, Kyoto, Japan; c The Clinical and Translational Research Center, University Hospital, Kyoto Prefectural University of Medicine, Kyoto, Japan

**Keywords:** focal therapy, Gleason score, localized prostate cancer, microwave tissue coagulation, PI-RADS

## Abstract

**Introduction:**

Focal therapy (FT) for localized prostate cancer (PC) aims to achieve cancer control and maintain quality of life. Microwave tissue coagulation (MTC) is a tissue-coagulation thermotherapy that has been used to treat solid tumors such as kidney, liver, and lung tumors. However, the use of this technology in lesion-targeted FT for PC has not been established.

**Methods:**

We will perform a prospective multi-center, single-arm, clinical study to evaluate the efficacy and safety of lesion-targeted focal MTC for localized PC. In eight centers with expert urologists for performing magnetic resonance imaging (MRI)/ultrasound (US) fusion biopsy, patients will be evaluated prospectively after lesion-targeted MTC. The target sample size is 65. This study was registered with the Japan Registry of Clinical Trials and ClinicalTrials.gov. Inclusion criteria were patients who had a single MRI-visible lesion with Prostate Imaging Reporting and Data System (PI-RADS) category 3 or 4 that was proven as Gleason score of 7 or 8 cancer by enrollment biopsy or patients who had a single MRI-visible lesion with PI-RADS category 4 or 5 that was proven as Gleason score of 6 or 7 by enrollment biopsy. The primary endpoint is the disappearance of the targeted cancer lesion at 6 months after microwave coagulation, evaluated by a combined response in prostate-specific antigen, MRI, and prostate biopsy.

**Discussion:**

The importance of this clinical trial is to establish a new ablative treatment option for lesion-targeted FT in PC.

## Introduction

For the treatment of localized prostate cancer (PC), treating the whole-prostate-gland, such as with radical prostatectomy and radiation therapy, has been the gold standard. However, such current standard treatment likely causes treatment-related adverse events (AEs) including incontinence and erectile dysfunction. Whole-prostate-gland treatment could be considered as overtreatment in a certain subset of patients with relatively low-risk PC^[1,2]^. Because the prognosis for localized PC is generally longer, maintaining pre-operative quality of life (QOL) is also important for patients. To avoid both overtreatment and undertreatment of localized PC, a novel therapeutic strategy of lesion-targeted focal therapy (FT) to target only clinically significant cancer (CSCa) with organ preservation has been developed with the aim of achieving both cancer control and maintaining QOL^[3,4]^. Although technical therapeutic modalities for FT of PC include cryosurgery, high-intensity focused ultrasound (HIFU), brachytherapy, irreversible electroporation, and photodynamic therapy, the efficacy of microwave tissue coagulation (MTC) has yet to be established.

**HIGHLIGHTS**

- For localized prostate cancer (PC), lesion-targeted focal therapy (FT) to target only clinically significant cancer with organ preservation has been developed with the aim of achieving both cancer control and maintaining quality of life.
- Microwave tissue coagulation (MTC) is a tissue-coagulation thermotherapy and has been used to treat solid tumors, including the kidney, liver, and lung. The efficacy of MTC for FT of localized PC has yet to be established.
- We will perform a prospective multi-center, single-arm, clinical study to evaluate the efficacy and safety of lesion-targeted focal MTC for localized PC. The primary endpoint is the disappearance of the targeted cancer lesion at 6 months after microwave coagulation, evaluated by a combined response in prostate-specific antigen, magnetic resonance imaging, and prostate biopsy.

MTC is a tissue-coagulation thermotherapy that induces cell-coagulative necrosis through damage to cellular and intracellular structure membranes while also denaturing and coagulating structural proteins and eradicating the local blood supply. This technique has been used to treat solid tumors, including the kidney, liver, and lung^[5–7]^. Moreover, microwave ablation enhances tumor-specific immune response in patients with hepatocellular carcinoma^[8]^. Immunotherapy is the current focus of research in the field of oncology due to a series of breakthroughs in this field^[9]^. We applied this method for lesion-targeted FT for localized PC and performed an exploratory pilot clinical trial with five patients and a 6-month follow-up period that found it was feasible with potentially safe and effective^[10,11]^.

The primary aim of this study is to assess the efficacy of MTC in cancer control of a magnetic resonance imaging (MRI)-visible, single PC target, compared to the reported data from radical prostatectomy as a standard surgical historical control. The secondary aim is to demonstrate the safety and superiority of MTC in preserving QOL, including sexual function and urinary continence, compared to the historical control.

## Material and methods

### Study design

We will perform a prospective multi-center, single-arm clinical study to evaluate the efficacy of focal microwave tissue coagulation for MRI-visible localized PC. In eight hospitals in which there are expert urologists for MRI/US-fusion biopsy, the patients will be evaluated prospectively after lesion-targeted MTC-FT.

### Study approval

The clinical trial is conducted as specified clinical research under the Japanese Clinical Trials Act (Act No. 16 of 14 April 2017), after receiving central review and approval from a certified review board (CRB). This study was also conducted in accordance with the International Council for Harmonisation of Technical Requirements for Pharmaceuticals for Human Use Good Clinical Practice (ICH-GCP), carried out as “Advanced Medical Care B” under the Japanese “advanced medical care” system, with notification to the Ministry of Health, Labour and Welfare (MHLW). “Advanced medical care” is defined as medical treatment using advanced medical technology as specified by the MHLW and other medical treatments that require evaluation from the perspective of ensuring the efficient provision of appropriate medical care to determine whether they should be covered by insurance benefits. To ensure effectiveness and safety, certain facility standards are set for each medical technology, and insurance medical institutions that meet these standards can use these technologies in combination with insurance medical treatment by submitting a notification.

The trial protocol received central ethical approval from the CRB of our institution. A notification of the trial’s commencement was submitted to the MHLW and was accepted. This trial was registered with the Japan Registry of Clinical Trials and ClinicalTrials.gov. Written informed consent will be obtained from all patients before trial registration according to the Declaration of Helsinki and the Japanese Clinical Trials Act. Independent monitoring and auditing will be planned according to the law.

**Figure 1.**
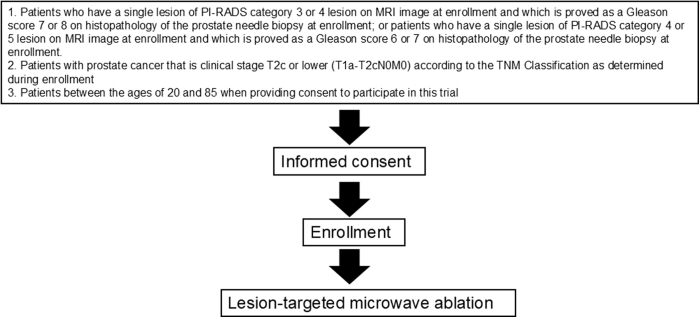
Study flow chart.

The flowchart of the study is shown in Fig. 1.

### Trial entry

All patients who have a newly diagnosed MRI-visible, targeted-biopsy-proven CSCa lesion will be counseled about the standard treatment option with curative intention (such as radical prostatectomy and radiation therapy). The basics of MTC and the current Japanese situation regarding FT for localized PC will be discussed with the patient.

### Subjects (patient eligibility)

In this clinical trial, a lesion to be targeted by MTC was defined as CSCa present in the prostate, referred to as the target lesion. Eligibility criteria were determined based on Prostate Imaging Reporting and Data System version 2.1 (PI-RADS) in MRI and the Gleason score in pathological diagnosis. As PC has been shown to occur spatially and temporally multiple times, clinically insignificant cancers (CISCa), which require continued observation through active surveillance, were referred to as non-target lesions. The presence of up to three CISCa lesions was permitted at the time of clinical trial registration.

The inclusion criteria are as follows:

1. Patients who had a single MRI-visible lesion with PI-RADS category 3 or 4 that was proven as Gleason score 7 or 8 by enrollment biopsy or patients who had a single MRI-visible lesion with PI-RADS category 4 or 5 that was proven as Gleason score 6 or 7 by enrollment biopsy (Table 1).
2. Patients with PC that is clinical stage T2c or lower (T1a–T2cN0M0), according to the TNM Classification as determined during enrollment.
3. Patients between 20 and 85 years old when providing consent to participate in this trial.
4. Patients from whom consent is obtained prior to enrollment.

**Table 1:** Inclusion criteria according to MRI and pathology.

	MRI		
	PI-RADS 3	PI-RADS 4	PI-RADS 5
Pathology
Gleason 6	X	○	○
Gleason 7	○	○	○
Gleason 8	○	○	X

The exclusion criteria are as follows:

1. Patients have a lesion identified as PI-RADS category 4 or 5 on MRI at enrollment, which is defined as a Gleason score of 6 and a diameter of less than 10 mm (the diameter of the lesion is defined as the longer of the lesion diameters identified on either MRI at enrollment or the tumor length as measured on histopathological examination of an enrollment biopsy).
2. Patients to have four or more non-target lesions. Non-target lesions are defined as the lesions defined in exclusion criterion 1 or lesions with a PI-RADS category 3 on MRI at enrollment and a Gleason score of 6 on biopsy at enrollment.
3. Patients to have a lesion with a PI-RADS category 5 on MRI at enrollment and a Gleason score of 8 on histopathology of prostate needle biopsy at enrollment (such lesions are referred to as “excluded lesions”).
4. Patients with a serum prostate-specific antigen (PSA) level over 20 ng/ml during enrollment.
5. Patients in whom the distance from the target PC lesion to the rectum is 10 mm or less on MRI (coronal or sagittal) obtained during enrollment.
6. Patients who have received an antiandrogen for benign prostatic hyperplasia prior to enrollment.
7. Patients who have received an antiandrogen for benign prostatic hyperplasia prior to enrollment.
8. Patients who have undergone surgery, drug therapy, or radiation therapy for PC prior to enrollment.
9. Patients with active multiple cancers.
10. Patients who wear a pacemaker.
11. Patients for whom MRI scans are contraindicated.
12. Patients in whom transrectal ultrasound (TRUS) cannot be performed such as for a constricted rectum.
13. Patients with a prothrombin time <50% or platelet count <60 000/mm^3^ during enrollment.
14. Patients deemed to be ineligible by an investigator.

### Intervention

The intervention will be performed according to previously published methods^[10]^.

#### Diagnosis with MRI and MRI/ultrasound (US)-fusion biopsy

At diagnosis before enrollment, MRI will be performed with a 3 T/1.5 T-MRI unit with a multichannel phased-array coil. The patients will undergo an MRI/US-fusion targeted biopsy. At least two biopsy cores will be obtained from each suspected lesion detected on MRI using Urostation® or Trinity®. In the same session after a targeted biopsy has been performed, a systematic biopsy, the result of which is required no CSCa in out-of-field for the MRI-visible target lesion, will be conducted. MRI/US-fusion targeted biopsies will be performed using a computer-based image-fusion system of Urostation® or Trinity® (Koelis, Meylan, France), which are three-dimensional (3D) US-based organ-tracking systems. These systems can be used to display the biopsy-needle or ablative-needle trajectories on 3D MRI/US-fusion imaging including the target lesion, which makes it possible to ensure that the needles is correctly targeted using 3D digitalized technology.

In this study, patient selection is based on the locations of the cancer-containing biopsy map according to MRI/US-fusion including an MRI-visible target lesion. Cross-sectional images of the prostate, including the 3D cancer map, will be prepared from 3 to 5 mm slices using Urostation® or Trinity®.

#### Lesion-targeted FT with MTC (Fig. 2a)

**Figure 2.**
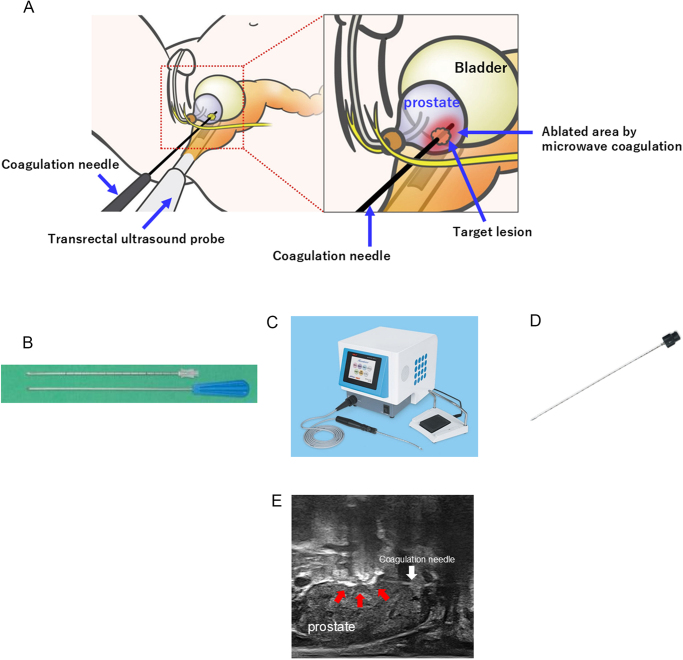
Microwave ablation. With the patient in the lithotomy position, one coagulation needle will be inserted into the target lesion via the transperineal approach using free-hand technique. Under biplanar transrectal ultrasound guidance using general versatile ultrasound equipment. (A) The guiding needle (14 G, 195 mm in length) (including external and internal needles) will be inserted just before the target lesion. After removing the inner needle, the coagulation needle will be inserted along the outer needle and punctured into the target lesion. (B) Microtaze® AFM-712 will be used for microwave ablation. This device utilizes the dielectric heat generated in the tissue by focused irradiation of 2450 MHz microwaves into the tissue. (C) The coagulation needle (1.6 mm in diameter, 250 mm in length) will be inserted through the guidance needle. (D) During coagulation, monitored with a real-time biplanar ultrasound probe, the bubbles generated by the high temperature of the microwave-ablated area will be visible on the ultrasound image as a high-echo area (red arrows).

The ablative treatment will be performed by trained urologists and supervised by an expert surgeon (OU) of lesion-targeted FT. On the day of treatment, patients will receive general or spinal anesthesia. With the patient in the lithotomy position, the Koelis system will be used to register the biopsy-proven cancer lesion using the 3D-TRUS probe. Under biplanar-TRUS guidance using a general versatile US equipment (Canon, a specific guiding needle (14 G, Alfresa Pharma Corporation, Osaka, Japan) (Fig. 2b) will be inserted into the target lesion via the transperineal approach using a free-hand technique^[12–14]^. The physician will interpret the 3D cancer maps based on a combination of both the MRI-visible lesion and positive biopsy trajectories for CSCa and then insert the guiding needle into the target lesion from perineal skin under the biplanar-TRUS guidance (using the general versatile ultrasound equipment) (Fig. 2a). Next, with the transrectal 3D-US probe (Koelis) switched from the biplanar transrectal 2D-US probe, the accuracy of 3D positioning for the guiding needle will be confirmed by MR/US image fusion technology using Urostation® or Trinity®. Microtaze® AFM-712 (Alfresa Pharma Corporation, Osaka, Japan) will be used as the MTC device (Fig. 2c). The coagulation needle (1.6 mm in diameter), which has the rugby-ball-shaped ablative tip with a maximum length of 20 mm and a maximum radius of 6 mm (Fig. 2d), will be inserted to the target through the guiding needle. In a therapeutic strategy, the estimated necrosis area with the coagulative needle should cover the target and approximately 5 mm distance from the margin of the target with consideration of safety margin. The output level of the MTC device will be set at 30 W, and the irradiation will be carried out for 60 s each. During MTC, in monitoring with a real-time biplanar-TRUS probe, the microbubbles generated in the high temperature of the MTC will be visible on the US image as a high-echo area (Fig. 2e). Intraoperative assessment of surgical technical success to demonstrate the effective ablation for the target will be performed by Doppler study using a biplanar-TRUS monitor to compare the presence or absence of blood flow signals in the target between intraoperative pre- and post-ablation. The operator will repeat the MTC until the target lesion plus peri-lesional area approximately 5 mm distance from the MRI-visible target margin will be completely ablated, resulting in the complete disappearance of the blood flow in these areas. A Foley catheter will be inserted immediately after ablation and removed between 6 and 24 hours after ablation in principle.

### Follow-up

The assessment schedule is shown in Table 2. The planned follow-up procedure consists of hospital visits at 10 days, 1, 3, and 6 months, during which the patients’ serum PSA levels will be measured and any AEs will be reported according to the Common Terminology Criteria for AEs (CTCAE, version 5). The patients will be asked to fill in Expanded Prostate Cancer Index (EPIC, Japanese version) questionnaires and Short Form Health Survey (SF)-12 at each hospital visit. Furthermore, the International Index of Erectile Function (IIEF)-15 and EuroQol 5 Dimension (EQ5D) will also be analyzed. Multiparametric MRI and a follow-up mandatory prostate biopsy will be scheduled for the 6-month follow-up examination.

**Table 2:** Follow-up schedule.

	Pre-treatment	Treatment	10 days Post-treatment	1 month	3 months	6 months	Withdrawal
Informed consent	○						
Questionnaires (EPIC, SF-12)	○		○	○	○	○	○
Questionnaires (EQ5D)	○					○	○
Questionnaires (IIEF-15)	○			○	○	○	○
PSA	○			○	○	○	○
MRI	○					○	○
Biopsy	○					○	○^a^
AEs							
Reason for withdrawal							○

^a^ If possible.

### Primary endpoint

Disappearance of the targeted cancer at 6 months after MTC, as evaluated by a combination of the responses in PSA, MRI, and prostate biopsy.

The primary endpoint is to achieve all of the following PSA, MRI, and biopsy criteria:

1. A 50% or greater reduction from the preoperative PSA level at postoperative 3 or 6 months.

2. A reduction of PI-RADS category of the targeted PC lesion to 3 or lower (including “difficult to judge” and “change after treatment”) on MRI at postoperative 6 months.

3. No cancer tissue detected in the histopathological examination of the targeted biopsy from the targeted cancer lesion performed 6 months postoperatively.

### Secondary endpoints

#### The secondary endpoint 1: serum marker, imaging, and histopathological indices

The secondary endpoint 1 is to achieve all of the following serum marker (1-A or 1-B), imaging (2-A or 2-B), and histopathological (3-A or 3-B) indices:

1-A: A 50% or greater reduction from the preoperative PSA level at postoperative 3 or 6 months.

1-B: A 50% or greater reduction from the preoperative PSA level at postoperative 3 or 6 months, and PSA <4 ng/ml.

2-A: A reduction of PI-RADS category of the targeted PC lesion to 3 or lower (including “difficult to judge” and “change after treatment”) at MRI at postoperative 6 months

2-B: A reduction of PI-RADS category of the targeted PC lesion to 2 or lower (including “difficult to judge” and “change after treatment”) at MRI at postoperative 6 months.

3-A: No cancer tissue in histopathological examination from the targeted biopsy from the targeted cancer lesion performed at postoperative 6 months.

3-B: Gleason score 6 or lower in histopathological examination from the targeted biopsy from the targeted cancer lesion performed at postoperative 6 months.

#### The secondary endpoint 2: PSA level

The secondary endpoint 2 is to describe the change of PSA level over time (pre-treatment, 3 months post-treatment, and 6 months post-treatment).

#### The secondary endpoint 3: imaging and histopathological indices

The secondary endpoint 3 is to achieve both imaging (2-A or 2-B) and histopathological (3-A or 3-B) indices, below:

- 2-A: A reduction of PI-RADS category of the targeted PC lesion to 3 or lower (including “difficult to judge” and “change after treatment”) at MRI at postoperative 6 months.
- 2-B: A reduction of PI-RADS category of the targeted PC lesion to 2 or lower (including “difficult to judge” and “change after treatment”) at MRI at postoperative 6 months.
- 3-A: No cancer tissue in histopathological examination from the targeted biopsy from the targeted cancer lesion performed at postoperative 6 months.
- 3-B: Gleason score 6 or lower in histopathological examination from the targeted biopsy from the targeted cancer lesion performed at postoperative 6 months.

#### The secondary endpoint 4: patient-reported outcomes (PROs)

The secondary endpoint 4 is to describe the change of PROs, such as EPIC, SF-12, and EQ5D over time.

#### The secondary endpoint 5: safety assessment

The secondary endpoint 5 is to assess safety including the occurrence and severity of AEs and malfunctions. Malfunctions and AEs occurring within 6 months (or before withdrawal) will be assessed with severity classified according to the CTCAE, version 5. AEs will be evaluated by examination and interview by the physician and by patient report. The examination and interview will be conducted every day during hospitalization and on subsequent return visits after surgery (10 days, 1 month, 3 months, and 6 months after surgery), and any medical treatment outside of these scheduled dates will be reported and evaluated in the same manner. Expected AEs include puncture site abnormalities (hematoma, subcutaneous bleeding, *etc.*), urinary symptoms (frequency, urinary urgency, dysuria, *etc.*), increased residual urine or urinary retention, hematuria, and urinary tract infection.

Pad-free ratio based on potential treatment-related urinary incontinence rate at 6 months post-treatment (defined as achievement of 0 pad use) will also be assessed.

### Exploratory endpoint

The exploratory clinical endpoint is to describe changes in serum values of S2,3PSA% over time (pre-treatment, 10 days, 1, 3, and 6 months post-treatment)

### Sample size considerations

In this study, Bayesian sample size determination based on a prior predictive distribution was used^[15]^. A non-informative Beta^[1]^ prior was set for the analysis. The success probability of the study treatment (achieving the primary endpoint) was estimated at approximately 70% from a previous study^[14]^; thus, a degenerate distribution at 70% was set as the design prior distribution.

The non-inferiority margin was determined using a fixed-margin method. From reports on robot-assisted radical prostatectomy^[15–19]^, which is the historical control, the success probability of the standard treatment is estimated at 67.4% (95% confidence interval: 65.5%–69.4%). We chose the non-inferiority margin as (1 − 0.8) × 100% of 65.5%, which equals 13.1%. The study treatment will be considered as non-inferior to the standard treatment if the posterior probability for the success probability exceeding the target value of 54% (= 67.4% − 13.1%) is greater than 0.95. To achieve a Bayesian power of at least 0.8, 60 patients are required. Considering patients will be excluded from the analysis, the target sample size is set at 65.

### Statistical methods

For the primary endpoint, the posterior distribution and 95% credible interval of the posterior mean for the success probability of the study treatment will be estimated using Beta^[1]^ as the prior distribution. The posterior probability that the success probability exceeds the target value (54%) will also be calculated.

Changes in PSA, S2, 3PSA%, and PROs (EPIC, SF-12, EQ5D, and IIEF-15) over time will be analyzed by summarizing the levels or scores at each time point and performing Wilcoxon signed-rank test for the changes from pre-treatment at each time point. For safety assessment, we will summarize the incidence proportion and severity of AEs and malfunctions up to 6 months post-treatment. We will estimate incidence proportion and its 95% confidence intervals for urinary continence.

### Monitoring and audit

Regular monitoring will be performed by the external and independent Coordinating Centre (APO PLUS STATION CO. LTD., Tokyo, Japan). Audits will be regularly performed by independent staff of the company. The purpose of the monitoring and the audits is to contribute to ensuring patient safety and adherence to ICH-GCP. Storage of research-related files will be in a locked cabinet in Kyoto Prefectural University of Medicine.

## Discussion

This multi-center, single arm clinical trial has been designed to evaluate the safety and efficacy of lesion-targeted MTC-FT for ablating MRI-visible, targeted biopsy-proven CSCa lesions. The safety and feasibility of MTC for PC was first reported in 1999^[20]^. In 2001, a Phase I/II trial was conducted in patients with local recurrence after radiotherapy for PC^[21]^, which reported that the frequency of negative biopsy findings at 24 weeks was 64%. Further studies with small sample sizes have been reported^[22]^. Oderda, *et al* reported early functional outcomes lead to no significant changes in IPSS and IIEF-5 scores in 11 patients with single MRI-visible PC who underwent transperineal-targeted MTC^[23]^. Delongchamps, *et al* reported performing MTC-FT on 10 patients of low- to intermediate-risk cancer with a visible index tumor on MRI^[24]^. MRI showed total necrosis in eight patients; moreover, cancer was not observed in four patients on rebiopsy at 6 months. Our pilot study, which enrolled five patients for 6 months follow-up, reported safety and short-term efficacy on the oncological outcome^[10,11]^. In terms of functional outcome, the EPIC questionnaire confirmed preservation of urinary, bowel, and sexual function, resulting in very high patient-reported satisfaction. In terms of treatment efficacy in our pilot study, 6 months post-operative MRI confirmed MRI-visible targeted lesion disappearance in all five patients, and biochemically, PSA levels decreased to 43% of pre-operative PSA (20–75%) at post-operative 3 months and to 57% of pre-operative PSA (22–91%) at post-operative 6 months, resulting in the achievement of a significant decrease (decrease-rate over 50%) from pre-operative PSA level as a biochemical endpoint. Among five patients, one patient withdrew because of the incidence of newly detected CSCa in an untreated area (out of the field of the targeted lesion), which was recognized as an under-diagnosis of a pre-operative systematic biopsy from out of the field of the targeted lesion at study entry. There were no AEs ≥ Clavien-Dindo Grade 3 related to MTC during the 6-month follow-up period. Based on these outcomes in the pilot study, this prospective multi-center single-arm study aims to compare MTC to historical controls of the current standard surgery (radical prostatectomy) with respect to efficacy in cancer control and functional outcomes for targeting a single MRI-visible CSCa lesion of localized PC.

The definitions of the clinical criteria for therapeutic success in FT for PC are disputed. We defined the primary endpoint as a composite of outcomes from serum markers, imaging, and prostate biopsy of the ablated target, following the guidelines for clinical trials evaluating medical devices for FT of PC^[24]^. Regarding PSA, we defined a reduction rate of 50% from pre- and post-ablation as the cutoff value in this study. Importantly, FT involves treated target areas and untreated areas within the prostate, and serum PSA is not cancer-specific but also PSA can be elevated by inflammation or volume of remaining normal prostatic tissues. Therefore, when using PSA testing to assess treatment response, it is crucial to consider PSA levels in both the cancer lesion (targeted treated area) and the rest of the prostate gland, which may include inflamed or normal tissue. Since the targeted FT treats a limited smaller area (target) than the larger untreated area (out of target), the targeted FT technique may have less impact on PSA than a hemiablation (half-gland ablation) FT technique. PSA nadir had been proposed as a useful post-FT tool^[26,27]^; however, its value is highly influenced by several factors, including preoperative PSA, prostate volume, presence of inflammation, and prostatic tissue ablation during treatment. In a retrospective study of a multi-center cohort of 703 patients (median follow-up 41 months) receiving FT, “PSA reduction rate” was found to be a surrogate predictor of the need for additional or salvage treatment. In addition, “PSA reduction rate” has been reported to be an independent variable predicting a positive needle biopsy for CSCa and any cancer after treatment^[28]^. Stabile, *et al* found that the probability of finding CPCa within 5 years from FT decreased for a %PSA reduction of >50%. They concluded that a %PSA reduction of at least 50% should be considered a proxy for good treatment quality and efficacy, providing a reduction in the probability of receiving either an additional treatment or a radical treatment within 5 years from treatment that remains stable until a %PSA reduction of 80%.

Regarding functional outcome for urinary incontinence, continence is defined as 0 pad use in our study. However, in many studies of radical prostatectomy, the definition of continence was defined as one or less pad use per day^[29,30]^. Importantly, even a small amount of incontinence has a negative impact on patient QOL^[31]^. Hence, we define no pad use as continence in this study. Regarding sexual function, targeted FT in general results in less effect on erectile function than whole-prostate-gland ablation^[32]^, probably because targeted FT likely does not affect erectile nerves. It should be noted that most previous studies focused on erectile function and overlooked other elements of male sexual function such as orgasm, ejaculatory function, and sexual desire^[33,34]^. Historically, there is a paucity discussion of ejaculation preservation following prostatectomy that removes the prostate, which creates prostatic fluid. As maintaining ejaculation function by FT likely positively impacts patient satisfaction, this study will evaluate these elements of sexual function using IIEF-15 questionnaires.

Although as the modality of FT, HIFU and cryoablation have been widely used^[12,35]^, the installation cost of such modalities is high. In contrast, as the installation cost of MTC is relatively lower, it is considered easier to install. In the coming higher-aging society, the number of elderly patients with PC is expected to increase; thus, minimally invasive and less expensive options that offer better QOL outcomes will be in demand. As such, it is conceivable that targeted FT with MTC is potentially a good option.

The recent development of artificial intelligence (AI) in medical research and medical diagnostic treatments provides valuable opportunities precisely for this purpose^[36,37]^. AI assistance can be expected to aid development in FT as well. MRI is very important in the diagnosis of the location of the PC within the prostate. This is particularly important for FT to target MRI-visible lesions. However, its reading requires expertise, and moreover, there is still no consensus protocol for the interpretation of MRI after FT. When AI becomes available for MRI evaluation, it will become a very powerful tool for generalizing FT. Furthermore, if AI can assist with needle placement during surgery, it is expected to reduce side effects and improve treatment outcomes through accurate targeted puncture. It is also expected to shorten the learning curve, which will be beneficial for the spread of lesion-targeted FT.

The limitations of this study would be the short follow-up period, relatively small sample size, and single-arm design. As to assess oncological outcomes, longer follow-up would be needed. We are going to plan another clinical protocol to report the safety and oncologic outcomes for a few years’ periods for the participating population of this study. This protocol is a single-arm design, which limits comparative conclusions against standard treatments, and randomized controlled trial (RCT) should be performed for scientific evidence. However, in focal treatment for PC, it is very difficult to perform RCT. Due to the low aggressiveness of low- and intermediate-risk PC, a randomized non-inferiority design that is powered on metastasis-free survival is simply not feasible. A trial like this would require >1000 patients and potentially 12–15 years of follow-up to reach maturity, which infers significant cost and commitment from physicians and patients. These issues have been highlighted by the premature closure of several trials testing new therapies for localized PC for reasons including cost, poor accrual, lack of physician equipoise, patient choice, and change of clinical practice^[38,39]^

## Conclusions

By combined assessment of pre- and post-treatment MRI, PSA level, and prostate biopsy, the MicroPro2 trial allows an appropriate evaluation of the efficacy and safety for percutaneously PC lesion-targeted MTC as prostatic functional preservation in a clinical setting. The outcome of this study would provide a new ablative technology option for lesion-targeted FT of localized PC.

## Data Availability

No datasets were generated or analyzed during the current study. All relevant data from this study will be made available upon study completion.

## Research registration unique identifying number (UIN)

This trial was registered with Japan Registry of Clinical Trials as jRCTs052240015 and ClinicalTrials.gov as NCT06430749.
